# 3-D Human Renal Tubular Organoids Generated from Urine-Derived Stem Cells for Nephrotoxicity Screening

**DOI:** 10.1021/acsbiomaterials.0c01468

**Published:** 2020-11-13

**Authors:** Haibin Guo, Nan Deng, Lei Dou, Huifen Ding, Tracy Criswell, Anthony Atala, Cristina M. Furdui, Yuanyuan Zhang

**Affiliations:** Wake Forest Institute for Regenerative Medicine, Wake Forest University Health Sciences, Winston-Salem, North Carolina 27157, United States; Reproductive Medicine, Henan Provincial People’s Hospital, Zhengzhou, Henan 450003, China; Wake Forest Institute for Regenerative Medicine, Wake Forest University Health Sciences, Winston-Salem, North Carolina 27157, United States; Department of Urology, Third Affiliated Hospital of Guangzhou Medical University, Guangzhou, Guangdong 510150, China; Wake Forest Institute for Regenerative Medicine, Wake Forest University Health Sciences, Winston-Salem, North Carolina 27157, United States; Department of Internal Medicine, Section on Molecular Medicine, Wake Forest University Health Sciences, Winston-Salem, North Carolina 27157, United States; Wake Forest Institute for Regenerative Medicine, Wake Forest University Health Sciences, Winston-Salem, North Carolina 27157, United States

**Keywords:** 3d culture, renal progenitor cells, kidney extracellular matrix, drug testing

## Abstract

The development of human cell-based systems to replace the use of rodents or the two-dimensional culture of cells for studying nephrotoxicity is urgently needed. Human urine-derived stem cells were differentiated into renal tubular epithelial cells in three-dimensional (3-D) culture after being induced by a kidney extracellular matrix. Levels of CYP2E1 and KIM-1 in 3-D organoids were significantly increased in response to acetone and cisplatin. This 3-D culture system provides an alternative tool for nephrotoxicity screening and research.

## Graphical Abstract

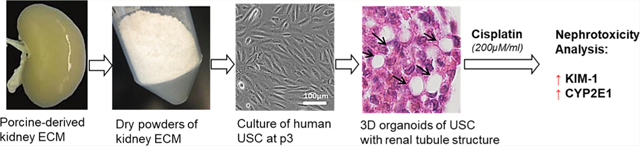

## INTRODCUTION

Acute tubular necrosis (ATN) is one of the most common causes of acute kidney injury. ATN is most frequently the result of the use of pharmaceutical compounds and substances such as heavy metals and organic solvents.^1^ It is extremely important to screen nephrotoxicity during drug development to accurately predict injury to the kidneys. Currently, there are two models that are used for testing drug-induced nephrotoxicity: rodent model and *in vitro* two-dimensional (2-D) culture of primary renal cells or renal cell lines. Rodents are often used as an *in vivo* model to evaluate the nephrotoxicity of new therapeutics; however, there are significant differences in the kidney that exist between rodents and humans, suggesting that rodent models are inadequate for human ATN research.^1^ Despite the predominant use of 2-D culture of renal tubular epithelial cells (RTECs), this model might not fully replicate both the morphology and physiology of three-dimensional (3-D) kidney tubules *in vivo* and fails to demonstrate appropriate injury responses to drug-induced nephrotoxicity *in vitro*. Cells in 2-D cultures have unlimited access to oxygen, nutrients, metabolites, and signaling molecules, which can result in over-sensitizing cultured cells to drug administration in contrast to the responses of the tissue *in vivo*.^2^ Thus, the development of relevant human cell-based drug screening systems is highly desired as a substitute to the use of rodent models and 2-D cultures in drug-related nephrotoxicity assessments.

Increasing evidence has demonstrated that *in vitro* 3-D organoids provide an alternative model for studying renal toxicology.^3^ Human RTECs are an optimal cell source for nephrotoxicity screens. Absorption of glucose, amino acids, drugs, and other substances from urine primarily occurs through the RTEC, which makes these cells vulnerable to drug-induced injury. Several cell types have been used for 3-D cultures, including immortalized kidney epithelial cell lines derived from human kidney (HK-2) or primary culture cells,^4,5^ pig kidney (LLC-PK1), dog kidney (MDCK),^6^ human urine cells,^5^ human embryonic stem cells (ESCs),^7,8^ or induced pluripotent stem cells (iPSCs).^7–12^ The different cell types, timeframes, and inductive factors are summarized in Table 1. RTECs are frequently used for early-stage safety assessment of large numbers of novel compounds, which facilitates the move away from *in vivo* rodent screening models toward less expensive and higher throughput cell-based assays.^13,14^ However, cell lines do not fully express all of the differentiated functions found in tissues *in vivo* because of the loss of appropriate cell polarity and tissue architecture, which can lead to changes in drug transporter expression.^15^ Although ESCs or iPSCs possess the potential for renal differentiation, the challenges of precisely differentiating these stem cells into the many functional cells found in the kidney limit their utility.^16^ Primary RTECs of human origin, being the predominant target of most toxicants in the kidney,^17,18^ are characterized by a defined cell polarity and the junctional assembly of epithelia, brush border enzyme activity, and metabolic and transport capacity.^19–21^ However, obtaining RTECs from a patient requires a kidney biopsy with the potential risks and complications associated with this invasive procedure.^22^ Thus, a novel stem cell source from the kidney for use in 3-D cultures, that is easy to isolate, expand, and differentiate, is highly desired for drug-related nephrotoxicity testing.

Our previous studies have demonstrated that human urine-derived stem cells (USCs) originate from the renal glomerular parietal stem cell population and possess self-renewal and multipotent differentiation capacity.^23,24^ USCs can be obtained *via* simple, safe, noninvasive, and low-cost approaches. About 3–7 USC clones per 100 mL urine can be isolated and one USC clone can generate one million cells at early passages (< p4) 4 weeks after isolation; therefore, USC clones can proliferate rapidly to produce the number of cells required for drug testing.

Differentiating stem cells to renal tubule epithelial cells has proven challenging despite extensive investigation. Our previous studies demonstrated that tissue-specific extracellular matrix (ECM) plays a major role in inducing and retaining the phenotypic characteristics of hepatocytes, cardiomyocytes, skeletal myocytes, and skin epithelial cells *in vitro*.^25–31^ The goal of this study was to determine whether porcine-derived kidney ECM (k-ECM)^32^ could induce human USC to differentiate into renal tubule epithelial cells that could form 3-D organoids to be used as a biological tool to test nephrotoxicity.

## RESULTS AND DISCUSSION

### Optimization of the 3-D Organoid Size.

Controlling the size of organoids is critical for the maintenance of maximum cell viability and the prevention of cell necrosis at the center of the spheres over time. The diameters of spheroids or organoids usually range from 200 to 500 *μ*m^33,34^ depending on the types of cells used and the length of culture. The optimal size of organoids formed with human USC was determined by seeding different numbers of cells and letting them grow for 1–2 weeks.

**Figure 1.**
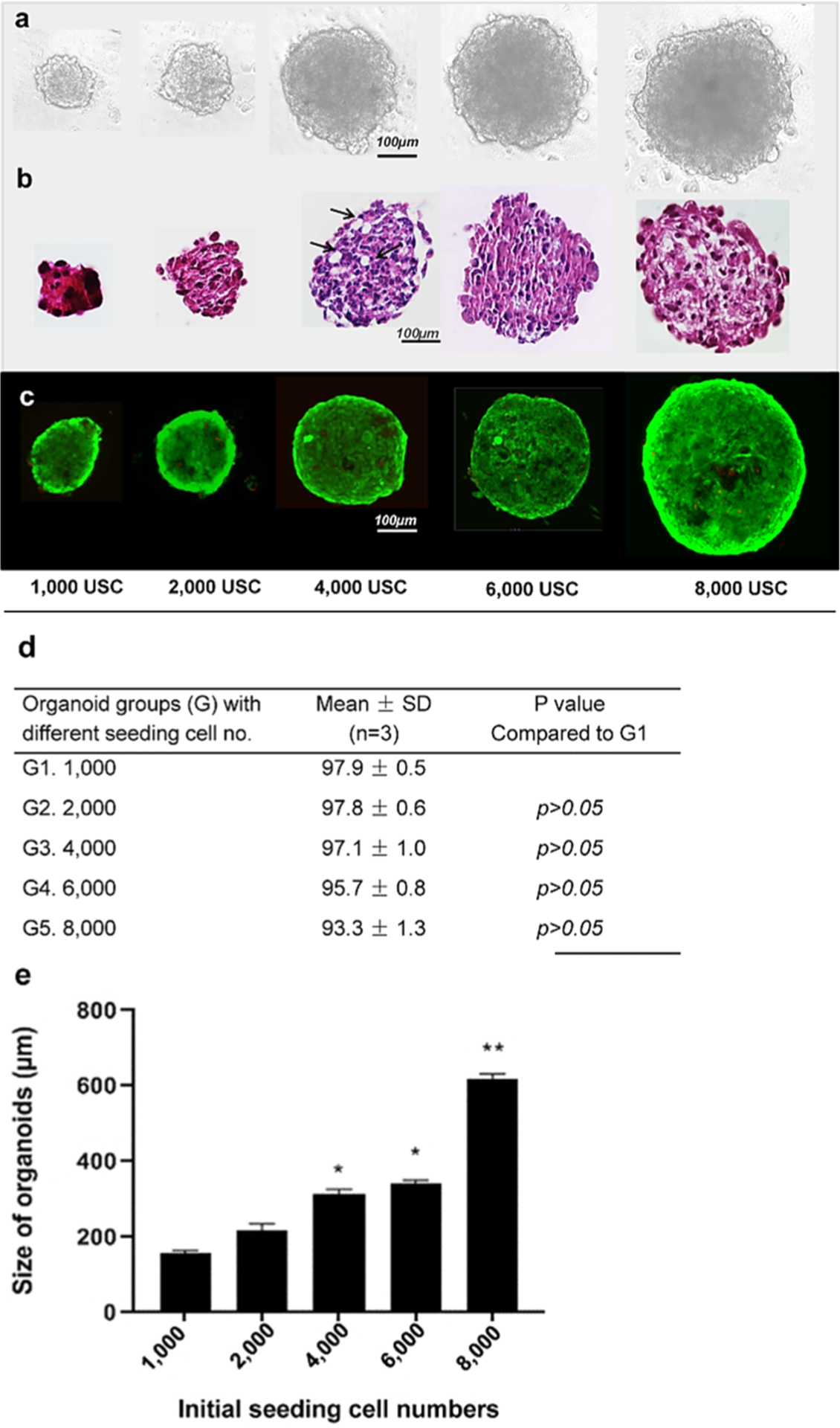
Viability of human USCs in 3-D organoids. (a) Initially seeded at increasing numbers of cells, after 1 week culture as assessed by bright-field microscopy; (b) H&E staining; (c) live/dead cell staining and fluorescent imaging; (d) cell viability of human USC 3-D organoids at different numbers of initial seeding cells one after 1 week culture, assessed by live/dead cell imaging assay; (e) Size of the 3-D organoids derived from human USC increased proportional with the numbers of cells initially seeded after 1 week culture.

**Figure 2.**
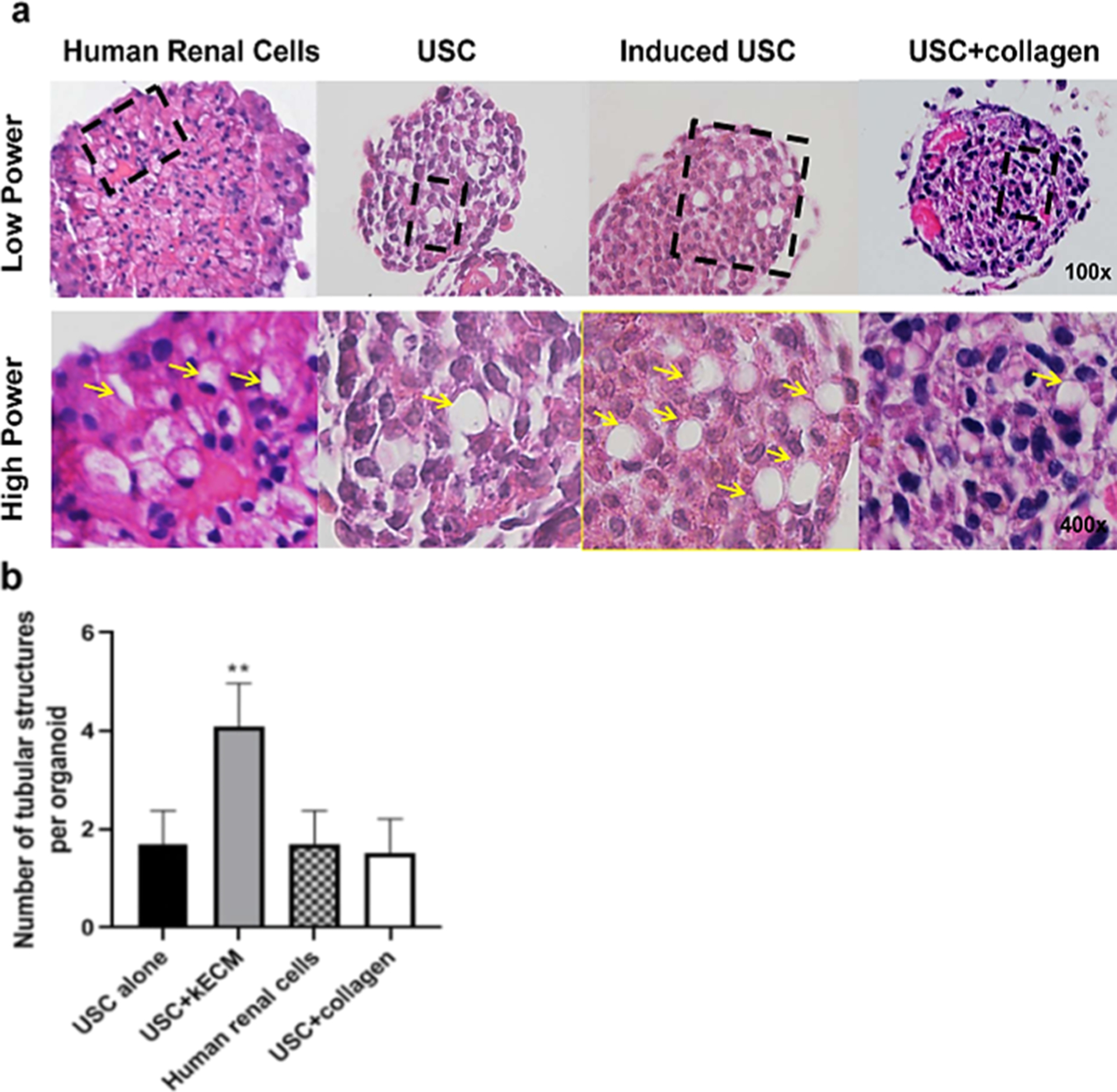
Tubular-like structure formation within the organoids after culture for 2 weeks. (a) Numbers of tubular-like structures significantly increased in the k-ECM-induced organoid group, compared to other three groups (*p* < 0.05); (b) comparison among three groups for tubular-like structures was performed by semiquantitative analysis. Data are presented as mean ± SEM. **p 0.01–0.05 (Student’s *t*-test). Renal organoids derived from USC with k-ECM.

Bioluminescence assays and ATP activity were used as determinants of cell viability (Figure 1). The ratio of live cells to total cell number within the organoids reached about 97% in organoids that began with 1000, 2000, and 4000 cells but decreased to 95 and 93% in organoids seeded with 6000 and 8000 after 7 days of culture. This indicated that organoids initially seeded with up to 4000 cells/well maintained higher cell viability over time. As expected, the size of the organoids increased proportionately with the number of cells initially seeded (Figure 1a–e) after 1 week in culture. The changes in the diameter of the organoids were as listed: 157 ± 14.2 *μ*m (1000 cells/well), 217 ± 16.3 *μ*m (2000 cells/well), 312 ± 11.5 *μ*m (4000 cells/well), 340 ± 9.7 *μ*m (6000 cells/well), and 616 ± 26.2 *μ*m (8000 cells/well) (Figure 1c). Importantly, primitive renal tubule structure formation was observed in the organoids that were started with 4000 cells (Figure 2a). Based on this data, we decided to use 4000 cells/well for the initial seeding density and an optimum organoid diameter of 312 ± 11.5 *μ*m for subsequent analyses.

The differentiation of a multipotent stem cell population into renal tubule epithelial cells remains challenging despite the existence of several published methods for this purpose including the use of conditioned medium from primary RTEC culture,^35^ the use of bone morphogenic protein-2/7 (BMP2/BMP7),^36^ and the use of fibroblast growth factor-9 (FGF9).^37^ Every tissue contains a combination of unique cell types and a tissue-specific ECM that provides the niche (microenvironment) for the optimal growth and differentiation of stem cells. Tissue-specific ECM has also been shown to support growth and differentiation of stem cells *in vitro*.^28,38^ Our previous studies demonstrated that tissue-specific ECM from skeletal muscle, skin, and liver tissues efficiently induced progenitor stem cells to differentiate into myogenic, dermal epithelial, and hepatogenic cell lineages, respectively.^27,28,38,39^

Kidney specific ECM (k-ECM) is a complex network of collagens, elastin, growth factors, and several glycoproteins and proteoglycans forming basal membranes and interstitial space.^32,40^ k-ECM functions, beyond providing a scaffold for cells, are increasingly being discovered including the guidance of stem cells to nephrogenic differentiation.^41^ The ECM composition is specific to the different functional segments of the nephron (glomeruli and renal tubules), which provides microenvironmental cues for cell growth and function, which cannot be replicated in traditional and universal culture conditions. In this study, we proposed that stem cell–matrix interactions would drive nephrogenic differentiation of USC in 3-D culture. We examined the ability of porcine-derived k-ECM to differentiate multipotent human USCs into renal cells within a 3-D culture environment as determined by the expression of key RTEC and podocyte makers.

Our previous studies^23,42,43^ demonstrated that there are several types of cells that are passed in the urine but only the stem cell populations can be expanded *in vitro*. Specifically, mature renal cells shed from the kidney in the urine cannot attach and grow on culture dishes. Rare renal cells have appeared in the primary culture (p0) but were subsequently washed away after initial passaging. In addition, in differentiated USCs, the induction process of differentiating USCs into renal cells occurred at the initiation of organoid formation through the addition of k-ECM into the 3-D culture. Previous studies also demonstrated that nondifferentiated USCs, or cells cultured in the absence of k-ECM, did not express podocyte or RTEC makers in 2-D culture.^24^ Therefore, nondifferentiated USC organoids and renal cell organoids served as negative and positive controls, respectively.

**Figure 3.**
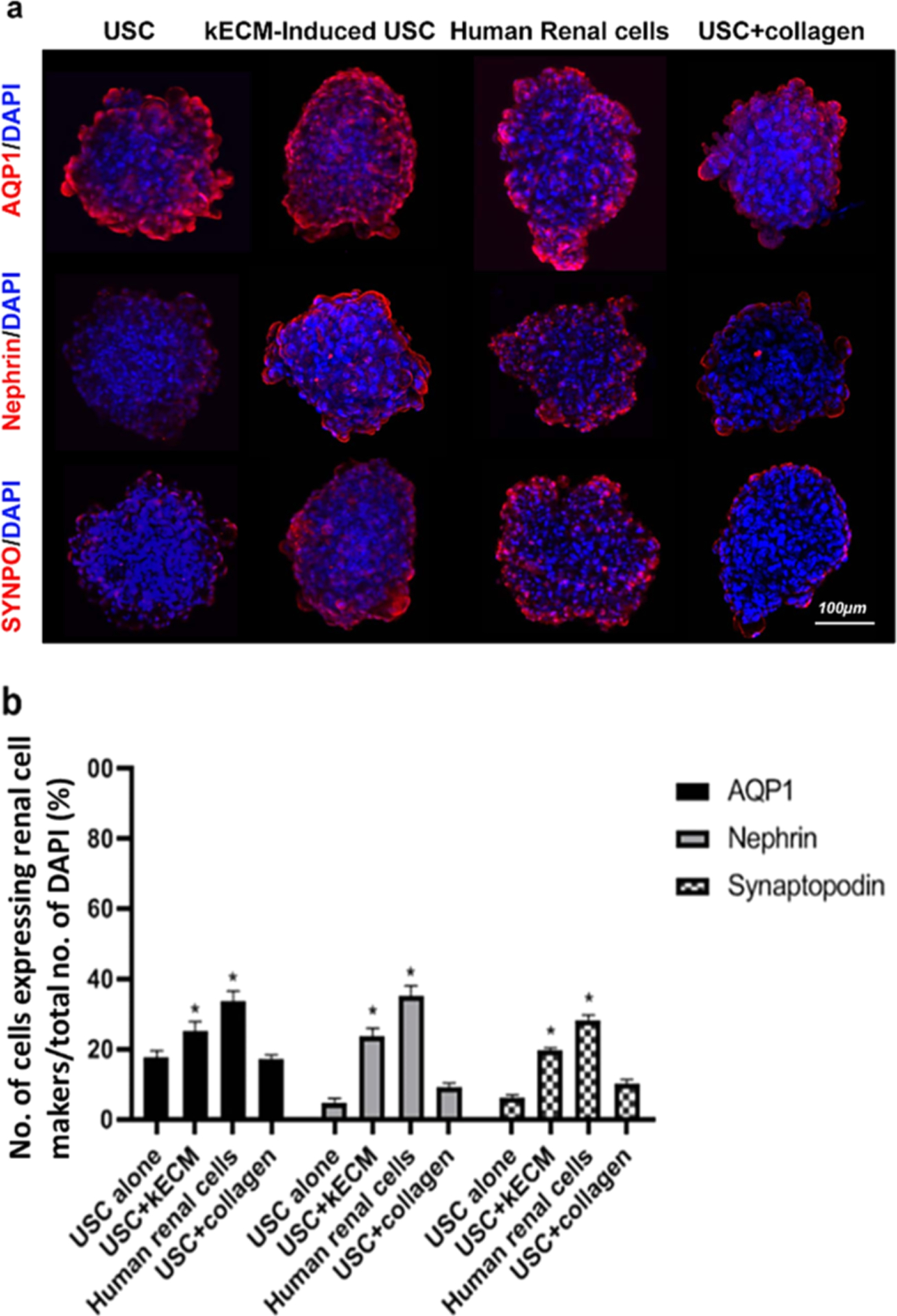
USC differentiated into RTEC in 3-D culture 14 days after k-ECM induction. (a) Proximal tubule epithelial cell marker (AQP1) and the podocyte markers (synaptopodin and nephrin) expressed in whole mounted organoids of reno-differentiated USC, compared to renal cells and USC organoids under a confocal microscope; (b) comparison among three groups for renal cell marker expression was performed by semiquantitative analysis. Data were quantified using ImageJ software (v1.52a, NIH, USA). Positive cells (%): percentage of positive immunolabeled cells over the total cells stained with DAPI in the entire section of each sphere. *p < 0.05, Student’s *t*-test.

To identify the potential of k-ECM to aid in the differentiation of USC into RTEC in 3-D organoid cultures, we compared USC grown in the presence of solubilized k-ECM gel (1 *μ*g/mL) (differentiated USC) at a ratio of 9:1 to USC without k-ECM (nondifferentiated USC)^44^ and collagen type as controls. H&E staining showed increased numbers of tubular-like structures within the organoids of USC grown with k-ECM as compared to those in the organoids without k-ECM or collagen type I (Figure 2a,b). In addition, immunostaining of whole mounted organoids demonstrated that expression of the proximal tubule epithelial cell marker (AQP1) and podocyte markers (synaptopodin and nephrin) were significantly higher in differentiated USC organoids as compared to those in nondifferentiated USC organoids with no k-ECM but significantly lower when compared to organoids derived from human renal cells (Figure 3a,b). Our data showed that k-ECM favors the enhanced renal tubular structure formation for drug-induced cytotoxicity, compared to the non-tissue-specific ECM control groups.

These data indicated that human USCs could differentiate into RTECs and podocyte-like cells within 3-D organoids and were able to form renal tubule-like structures in the presence of porcine-derived k-ECM.

Ideally, the ECM would come from the same species, which may offer better stem cell–matrix communication for the guidance of cell differentiation. Practically, animal-derived tissue-specific ECMs particularly porcine ECMs are easy to be obtained and often used as a matrix or scaffold in cell culture *in vitro* or tissue repair *in vivo*. The advantage of using porcine k-ECM is its compositional, structural, and molecular similarity to human k-ECM.^32^ It is also readily available in large amounts. Limitations include the potential loss of soluble growth factors and cytokines during the decellularization process and the heterogeneous composition of the ECM from batch to batch.

### Drug-Induced Nephrotoxicity.

Drug-induced nephrotoxicity is the primary cause of acute kidney injury resulting in a high incidence of morbidity and mortality.^45^ Therefore, it is extremely important to detect drug-induced nephrotoxicity early to protect long-term kidney function. *In vitro* models used to assess the nephrotoxicity of pharmaceutical agents usually rely on measures of cell death and cytoskeletal protein defects such as when cells are exposed to acetone and cisplatin,^3^ common agents used to induce nephrotoxicity.

Changes in the expression of the kidney injury marker molecule-1 (KIM-1) and the CYP450 protein CYP2E1 are commonly used to show the nephrotoxic effects of tested compounds. KIM-1 is a transmembrane protein expressed in dedifferentiated proximal renal tubular epithelial cells in damaged tissue and is often used as a marker of nephrotoxicity *in vitro*. The cytochrome P450-2E1 (CYP2E1) is an enzyme that is constitutively expressed in hepatic and extrahepatic tissues, including the kidney.^46^ As a common target for exogenous agent detoxification,^47^ CYP2E1 is an effective generator of reactive oxygen species and produces powerful oxidants (*i.e*., hydroxyl radical) in the presence of catalytic iron to induce cell injury.^46,48^

Cisplatin is a commonly used chemotherapeutic drug restricted in its clinical application because of severe nephrotoxicity.^49^ Cisplatin has previously been tested in 3-D organoid cultures but under nonphysiologically relevant conditions.^3,49^ Acetone is an industrial solvent that is relatively less toxic than many other solvents used; however, at high concentrations, acetone vapor can cause CNS depression, cardiorespiratory failure, and death.^50^ *In vitro*, acetone was shown to cause increased cytotoxicity in murine RTEC organoids.^3^ It is important to note that the dose required for cell toxicity, for both agents, is much higher *in vitro* than the dose that causes nephrotoxicity *in vivo*.

**Figure 4.**
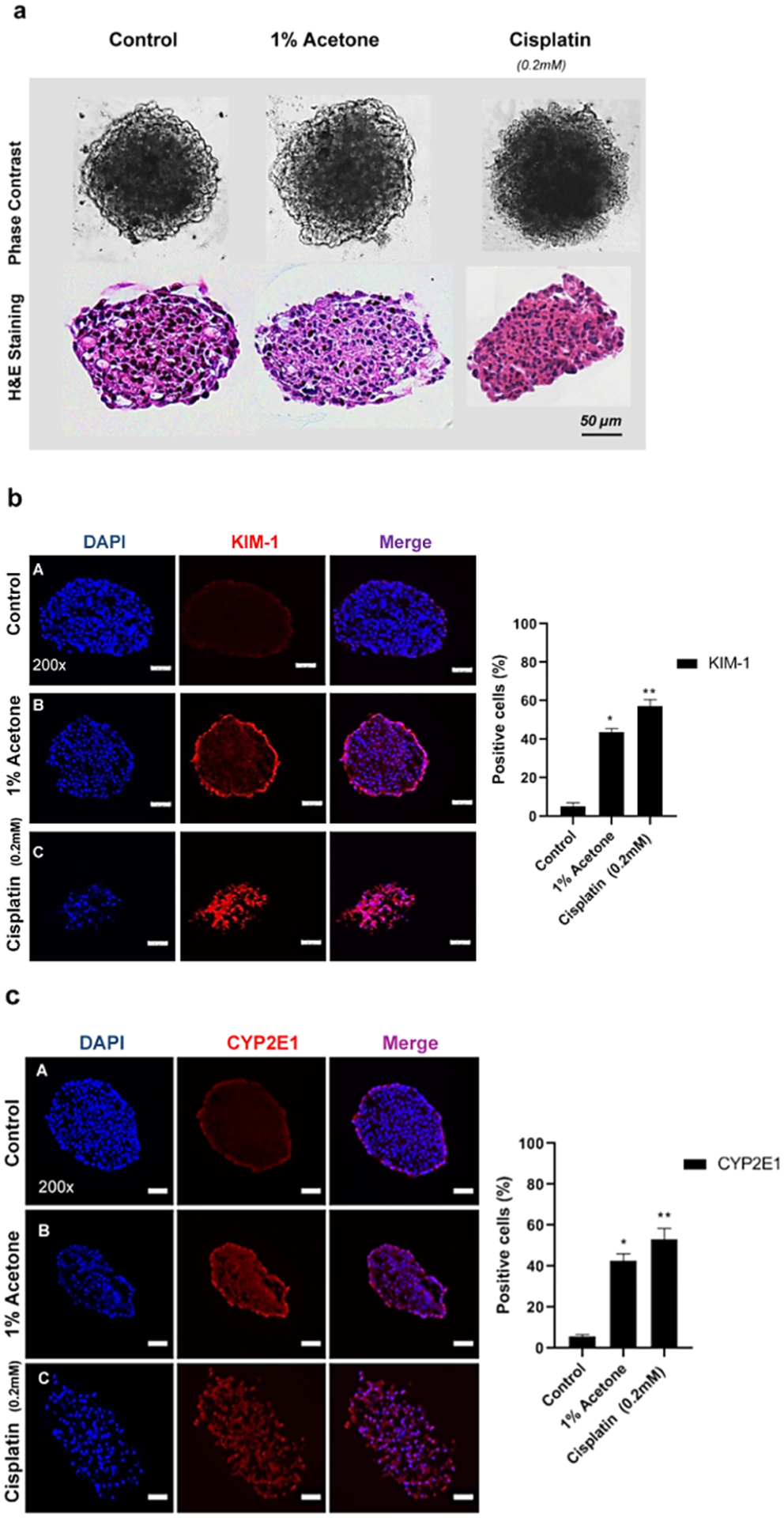
Drug-induced cytotoxicity test on differentiated USC (p3) 3-D renal organoids induced by k-ECM 3 days after exposure to acetone (1%) and cisplatin (0.2 mM), compared to control (k-ECM-induced organoids alone without any chemical reagent treatment). (a) Morphology of 3-D organoids treated with cisplatin and acetone, compared to control, under phrase contract and H&E staining; (b) expression of KIM-1 in cisplatin and acetone-treated organoids was significantly higher than control organoids. Cisplatin-treated organoids showed significantly higher levels of cytotoxicity than acetone-treated organoids. Fewer cells were left on the cross section stained slides of the cisplatin-treated organoids because of the dead cells being washed away during histological analysis processes; (c) expression of CYT2E1 in cisplatin-treated organoids was significantly increased as compared to the acetone-treated group, indicating that both treatments resulted in cytotoxicity, but that was more severe in the cisplatin-treated group. Positive cells (%): percentage of positive immunolabeled cells over the total cells (DAPI) in entire section of each sphere.

The ability of the 3-D culture to respond to physiologically relevant doses of nephrotoxic agents was determined by assessing changes in the expression of CYP2E1 and KIM-1.^3^ To test whether the renal organoids would respond to drug-induced toxicity, we treated the 3-D cultures of differentiated USC for 72 h with cisplatin (0.2 mM) or acetone (1%) as previously reported.^3^ These doses were toxic to most of the cells within the organoids assessed by the organoid morphology and the expression of KIM-1 and CYP450 3 days after exposure.^3^ Phase-contrast images of the 3-D renal organoids exposed to cisplatin showed a loss of semi-transparency at the edge and cloudy at the center of organoids that is indicative of dead cells. This is confirmed in H&E stained sections, showing that apoptotic cells decrease in size resulting in an increased nuclear/cytoplasmic ratio (Mean ± SD: 90% ± 6) (Figure 4a). Importantly, organoids were treated with cisplatin increased expression of CYP450 and KIM-1 that are usually not detected under nontoxic conditions.^3^ Immunofluorescence staining for KIM-1 (Figure 4b) and CYP2E1 (Figure 4c) expression in response to acetone and cisplatin showed the presence of both biomarkers in the exposed organoids.

## CONCLUSIONS

Human urine-derived stem cells induced to differentiate into RTECs by k-ECM formed 3-D renal tubular organoids, which were sensitive to the nephrotoxic agents: cisplatin and acetone. These organoids could provide promising tools for nephrotoxic drug screening as an alternative to animal models. Combined with the 3-D bioprinting technology, the use of primary human USCs could provide a novel high throughput screening tool for the identification of nephrotoxic agents early in the drug development process, prior to reaching the clinic. In addition, 3-D organoids using patient-derived USCs could provide a novel tool for kidney modeling and renal disease research in personalized medicine.

## FUTURE STUDIES

In future studies, we will perform additional analysis examining expression of ZO1, cadherin, and Na+−K+ ATPase to better identify the tubular structures generated. In addition, the ratio of podocyte and renal tubule cell marker expressions will be more accurately characterized by western-blot analyses and immunocytochemistry. Although K-ECM induced USCs to give rise to cells expressing both renal tubule epithelial cells and podocyte markers, we observed only tubule-like structure formation but not a glomerulus structure in the 3-D organoids after 2 weeks of culture. Histologically, glomeruli possess complex arrangements with at least four cell types (podocytes, endothelial, mesangial, and patriate cells) and the glomerular filtration barrier. It may take longer than 2 weeks of culture to form the more complex glomeruli. In contrast, renal tubules with mainly renal epithelial cells are relatively simple. This might be the reason that only renal tubular-like structures were observed. Further investigations are needed to study whether glomerulus structures can be generated in 3-D organoids. Importantly, more studies will be performed to determine dose- and time-dependent effects of the nephrotoxic chemicals on the USC-derived 3-D organoids prior to potential use in clinical cytotoxicity assessments.

## EXPERIMENTAL SECTION

### Culture of Human USCs.

All experiments were conducted under a protocol approved by the Wake Forest University Health Sciences Institutional Review Board for obtaining human tissues, including urine samples and kidney tissue from surgical waste materials for this study. To collect human USCs, fresh voided urine samples (100–200 mL) were obtained from three male healthy donors, 25–35 years of age. The urine samples were transferred to the laboratory for isolation and culture, as previously described.^23,42,43^ USCs were isolated and expanded in 24-well plates in mixed media composed of embryo fibroblast medium (EFM) and keratinocyte serum-free medium (KSFM) (1:1 ratio). When the clones reached 30% confluence after approximately 12 days (p0), the USCs were detached and transferred into a 6-well plate at 90% confluence (p1).

### Preparation of Kidney ECM.

Porcine kidneys were obtained from a local slaughterhouse or from surgical waste materials from animal surgeries. To generate k-ECM, kidneys were decellularized as described previously.^39^ Briefly, kidneys were rinsed in precooled Dulbecco phosphate-buffered saline and cut into small cubes (<1 cm^3^) after the removal of the attached connective tissues. The tissue fragments were frozen at −80 °C for 24 h. To remove the cellular components, the tissues were rinsed with double-distilled water (ddH_2_O) on a rotary shaker at 4 °C at 200 rpm for 3 days and then washed in 5% fetal bovine serum (v/v) (GE Healthcare, Logan, UT) for another 3 days. To rinse the tissue, the suspension solution was refreshed every 8 h. DNA concentrations were measured to confirm the removal of the cellular components. K-ECM was used experimentally when the DNA levels decreased to less than 10% of the original levels detected in the fresh kidney tissues, less than 50 ng DNA per mg tissue dry weight. K-ECM compositions were analyzed by the Quantibody Growth Factor Array, which detected a wide variety of growth factors and cytokines (amphiregulin 69.3 pg/mL, BDNF 1.8 pg/mL, bFGF 17.4 pg/mL, BMP-4 16.9 pg/mL, BMP-5 2812 pg/mL, BMP-7 1123.5 pg/mL, EGF receptor 2.7 pg/mL, FGF-7 20.7 pg/mL, Somatotropin 39.7 pg/mL, HB-EGF 5.7 pg/mL, IGFBP-1 3.2 pg/mL, IGFBP-2 8.7 pg/mL, IGFBP-4 528.9 pg/mL, IGFBP-6 125.5 pg/mL, NGF receptor 6.6 pg/mL, nt-4 3.2 pg/mL, TGF beta-1 778.8 pg/mL, VEGF receptor 2 13.3 pg/mL) in the ECM solutions as described in our previous study.^51^

### Fabrication of Kidney ECM Gel.

The lyophilized tissue samples were ground into fragments with a freezer mill (SPEX SamplePrep 6870 Freezer/Mill, Metuchen, NJ). One gram of ECM was mixed with 100 mg pepsin from porcine gastric mucosa containing 3,400 units of protein (Fisher Scientific, Fair Lawn, NJ) and sterilized by gamma irradiation (1 Mrad). The supernatant solution was neutralized with 0.1 N NaOH and stored at −80 °C until further use of neutralized solution (10 *μ*L) of each tissue, or ECM was measured using a NanoDrop spectrophotometer (NanoDrop 2000; Thermo Fisher Scientific, Waltham, MA).

### Optimization of the Organoid Size.

USCs at p3 were resuspended in medium and seeded into 96-well Clear Round Bottom Ultra Low Attachment Microplate (Corning, Individually Wrapped, with Lid, Sterile) at different cell densities: 1000, 2000, 4000, 6000, and 8000 cells/40 *μ*L drop volume at 37 °C in an atmosphere of 5% CO_2_. To determine the optimal size, the organoids were maintained in culture for 7 days. Half of the culture medium was removed and replaced with fresh medium every day. To induce USC differentiation into renal cells, 1 *μ*g/mL solubilized k-ECM was added into the culture medium at a ratio of 9:1 (culture medium: k-ECM gel) for 14 days.

### Measures of Cell Proliferation and Viability.

A Live/Dead cell viability kit (Molecular Probes) and ATP measurement assays (CellTiter-Glo Luminescent Cell Viability Assays, Promega, Madison, WI, USA) were used to assess cell proliferation and viability of the USC in the 3-D cultures. For the Live/Dead assay:^52^ USCs were cultured in a serum-free medium, and live cells and dead cells were differentiated by green or red fluorescence, respectively, and assessed using an Olympus FV10i confocal microscope. The number of viable cells was also determined based on the presence and quantity of ATP. The medium was discarded and the CellTiter-Glo 3-D reagent was added to each well. After incubating for 30 min at room temperature on a rotary shaker, the bioluminescence activity was assessed using a plate luminometer. Viability was monitored at 7 days.

### Histological Evaluation of Renal Organoids by Whole Mounting Staining.

After 14 days of culture, organoids were pooled and fixed in 4% paraformaldehyde (PFA) for 30 min at room temperature. Fixed organoids were embedded in Histone Gel and paraffin and sliced into 5 *μ*m sections. Deparaffinization and hematoxylin and eosin staining (H&E staining) was performed based on standard protocols. Organoid sections were imaged using a Zeiss Axiovert 200M microscope.

### Immunofluorescence Staining.

The morphologic characteristics of the renal organoids were observed using a phase-contrast microscope (Zeiss) and quantitated with ImageJ software (1.52a, NIH, USA). Whole organoids, or sections of organoids, after 14 days culture were fixed with 4% paraformaldehyde for 15 min at room temperature and blocked with serum-free block solution (Dako, Denmark) for an additional 15 min.

Four types of organoids were generated: nondifferentiated USCs (no k-ECM), differentiated USCs (with k-ECM), USCs with collagen type I (rat tail, 3.5 *μ*g/mL, Corning Inc, Corning, NY) or human renal cells as control. Fixed cells were incubated with primary antibodies to Aquaporin-1 (AQP1, L-19), Synaptopodin (SYN, P-19), and Nephrin (NEP1, B-12) overnight at 4 °C. Subsequently, cells were incubated with secondary antibodies (Vector Laboratories, Burlingame, CA, 1:200) for 1 h and mounted with antifade mounting medium containing DAPI (Vector Laboratories). Images were obtained using a fluorescence microscope (Leica DM 4000B, Germany) or a confocal microscope (Olympus FluoView Fv10i, Olympus Life Science, Shinjuku, Tokyo, Japan). Investigators who conducted the immuno-fluorescent analyses of the renal proteins were blinded to the experimental groups. Samples were assessed in triplicate. Both the numbers of stained cells per section and the percentage of the area stained compared to total area were calculated using ImageJ software (1.52a, NIH, USA).

### Analysis of Nephrotoxicity.

k-ECM-induced 3D organoids were divided into three groups for nephrotoxicity testing: acetone (1%), cisplatin (0.2 mM) treatment, and control (without chemical reagent treatment) for 3 days, respectively. Increased expression of CYP2E1 and KIM-1 are specific signatures of nephrotoxicity. The cytotoxicity of USC organoids exposed to cisplatin or acetone was assessed using a method described in a previous study,^3^ using immunofluorescence, as described above. Organoids were exposed to 1% acetone (Fisher, USA) or 200 *μ*mol/L cisplatin for 3 days. Cisplatin was stored as a 200 mM stock solution in dimethyl sulfoxide (DMSO) and diluted to its final concentration in PBS/media. The cisplatin was sterile filtered using a 0.22 mm filter prior to application to the organoids. After 3 days of drug exposure, the organoids were pooled and fixed in 4% PFA. Deparaffinization and H&E staining were performed as described above. Antibodies used: secondary Alexa-488 goat-anti-mouse IgG antibody (1:1000) (Invitrogen, USA), CYP2E1 antibody (1:1000) (Millipore, USA), and R9 KIM-1 antibody (1:500) (Merck & Co, Rahway, NJ, USA). All slides were imaged using an Olympus FV1200 Laser Scanning Confocal Microscope.

### Statistical Analysis.

All quantitative data analysis was used SPSS 20.0 (IBM, 2011). Means of the three independent experiments were compared in a nonpaired two-tailed *t*-test, or a one-way ANOVA was performed, combined with Tukey post hoc tests. *P* < 0.05 was considered statistically significant.

## Supplementary Material

Supplementary material

## Figures and Tables

**Table 1.** Summary of *In Vitro* Nephrogenic Differentiation of Stem Cells in 3D Culture^a^

authors, year, and citation	cell types	3D culture patterns	induction factors	induction time frame (days)	outcomes and application	advantages and limitations
Taguchi *et al.* cell stem cell 2014^11^	hiPSC → NPC	air–liquid interface culture	mouse embryonic spinal cord	6	kidney organoids of hiPSC, containing nephron-like structures	+ part of nephron-like structure
						− no entire nephron structure
Morizane *et al.* nature biotechnology 2015^7^	hESC/hiPSC → NPC	ultralow attachment culture	FGF9 plus CHIR	14	nephrotoxicity test (cisplatin)	above
Freedman *et al.* nature communications 2015^8^	hESC	matrigel sandwich	CHIR	16	nephrotoxicity test (cisplatin)	above
Takasato *et al.* nature, 2015^12^	hiPSC	transwell 0.4 *μ*m pore polyester membrane	FGF9 plus CHIR and heparin	11–18	nephrotoxicity test (cisplatin)	above
Schutgens *et al.* nature biotechnology 2019^5^	h/m kidney cells or UC	matrigel	FGF-10, rhokinase inhibitor Y-27632, A8301	6	tubuloids from patients with cystic fibrosis	above

a Abbreviations: hiPSC: human-induced pluripotent stem cells; hESC: human embryonic stem cells, NPC: nephron progenitor cells; UC; urine cells.
