# Supplementary material for: 3-D Human Renal Tubular Organoids Generated from Urine-Derived Stem Cells for Nephrotoxicity Screening
Source: ACS Biomater Sci Eng. Author manuscript; Available in PMC 2021 May 13. (PMC8118570; doi:10.1021/acsbiomaterials.0c01468)
Supplement: Supplementary material [file NIHMS1701599-supplement-Supplementary_material.pdf]

3-D Human Renal Tubular Organoids Generated from Urine-Derived Stem Cells for Nephrotoxicity Screening

Haibin Guo∇, Nan Deng∇, Lei Dou, Huifen Ding, Tracy Criswell, Anthony Atala, Cristina M. Furdui and Yuanyuan Zhang

Number of pages: 9

Number of figures: 4

Number of tables: 1

ASSOCIATED CONTENT

Supporting Information.

Figure Legends

Figure S1. Optimization of 3D organoid size and cell viability. a) The size of the organoids increased with increased seeding density (from 1,000 to 8,000 cells) after 7 days of culture as viewed using phase-contrast microscopy (scale bar 100 μm); b) Paraffin sections of 3D organoids were stained with H&E for internal morphologic characterization. Tubular-like structure formation in organoids with 4,000 cells (arrows); c) Organoids were stained using Life Technologies LIVE/DEAD Cell Imaging Kit (live cells = green, dead cells = red) and imaged using an Olympus FV10i confocal microscope (bottom panels, I–IV). Scale bars represent 100 μm; d) Cell Viability of human USC 3D organoids at different numbers of initial seeding cells one after week culture, assessed by Live/Dead cell imaging kit e) Quantification of organoid size over time (n = 6). Data presented as mean ± SD. Significance: $*p < 0.05$.

Figure S2. Tubular structure formation within organoids composed of USCs two weeks after exposure to k-ECM. **a**). Paraffin sections of USC-derived organoids (arrows) stained by H&E (low and high magnification); **b**). Quantification of tubular structure formation in 3D culture (n = 6). Data presented as mean ± SD. Significance: $*p < 0.05$.

Figure S3. USCs exposed to k-ECM were differentiated into renal cells after 14 days in 3D culture. **a**). Confocal images of human USC derived organoids (scale bar, 100 μm). Induced USC (4,000 cells at *p3*) expressed the renal tubular epithelial cell marker (AQP1) and podocyte markers (nephrin and synaptopodin, *i.e.* SYNPO); **b**). Quantification of cells that expressed renal cell markers in 3D culture (n = 6). Data presented as mean ± SD. Significance: $*p < 0.05$.

Figure S4. Cisplatin and acetone-induced cytotoxicity of induced-USC (*p3*) organoids 3 days after treatment. **a**). USCs in 3D culture displayed semi-transparency around the edges of organoids as seen by phase contrast microscopy and cell structure was well retained as seen by H&E staining (left column) (scale bar, 50 μm). Organoids showed signs of apoptosis or necrosis when exposed to 1% acetone. About 2/3 of the cells displayed some degree of nuclear dissolution (karyolysis) and/or nuclear fragmentation in H&E stained sections (middle column). Almost all cells in the 3D organoids appeared dark with loss of the semi-transparency seen in untreated organoids by phrase contrast microscopy. Nuclei condensation or pyknosis indicated cell death or necrosis when exposed to cisplatin (200 μm/ml) (right column); **b**). KIM-1 and **c**). CYP2E1 expression following drug exposure to cisplatin and acetone for 3 days (n = 6). Data in graphs presented as mean ± SD.

AUTHOR INFORMATION

Corresponding Author

*Phone: 1-336-713-1189 (office).

E-mail: yzhang@wakehealth.edu

ORCID

Yuanyuan Zhang: 0000-0002-0050-0051

Author Contributions

∇Haibin Guo, and Nan Deng contributed equally to this work.

Notes

The authors declare no competing financial interest.

ACKNOWLEDGMENT

The authors appreciate the financial support from the CKD Biomarkers Consortium Pilot and Feasibility Studies Program funded by the National Institute of Diabetes and Digestive and Kidney Diseases (NIDDK: U01 DK103225) and from National Institute of Allergy and Infectious Diseases (NIAID R21 AI157876).

ABBREVIATIONS

ATN, Acute tubular necrosis; RTEC, Renal tubular epithelial cells; USC, human urine-derived stem cell; k-ECM, kidney extracellular matrix; SYNPO, synaptopodin; KIM-1, kidney injury markers molecule-1; KSFM, keratinocyte serum free medium; DPBS, Dulbecco phosphate-buffered saline; ddH2O, double-distilled water; PFA, paraformaldehyde; H&E staining, hematoxylin-eosin staining.
